# Supplementary material for: Exploring the Importance of Environmental Complexity for Newly Hatched Zebrafish
Source: Animals (Basel). 2024 Mar 28;14(7):1031. doi: 10.3390/ani14071031 (PMC11011065; doi:10.3390/ani14071031)
Supplement: Supplementary file 1 [file animals-14-01031-s001.zip › Supplementary materials.pdf]

Table S1. Hole diameters and size ratios used in this study (Experiment 1) and in the previous one [33].

Diameter of larger hole	Diameter of smaller hole	Ratio between the areas
0.8 cm	0.62 cm	0.60
0.8 cm	0.69 cm	0.75
0.8 cm	0.74 cm	0.86
0.8 cm	0.76 cm	0.91

Table S2. Discrimination performance of the two treatments in Experiment 1. The table shows the proportion of passages through the larger hole, 95% Confidence Interval and binomial tests for all ratios and for both treatments.

Treatment	Ratio	Proportion of choices for the larger hole (mean ± SD)	95% Confidence Interval	Binomial Tests
Control	0.60	0.752 ± 0.124	0.676, 0.828	$p < 0.001$
	0.75	0.648 ± 0.075	0.595, 0.700	$p < 0.001$
	0.86	0.601 ± 0.066	0.518, 0.685	$p < 0.01$
	0.91	0.588 ± 0.050	0.531, 0.645	$p < 0.001$
Enrichment	0.60	0.750 ± 0.137	0.678, 0.822	$p < 0.001$
	0.75	0.615 ± 0.075	0.562, 0.668	$p < 0.001$
	0.86	0.575 ± 0.077	0.528, 0.622	$p < 0.001$
	0.91	0.583 ± 0.104	0.457, 0.709	$p < 0.01$

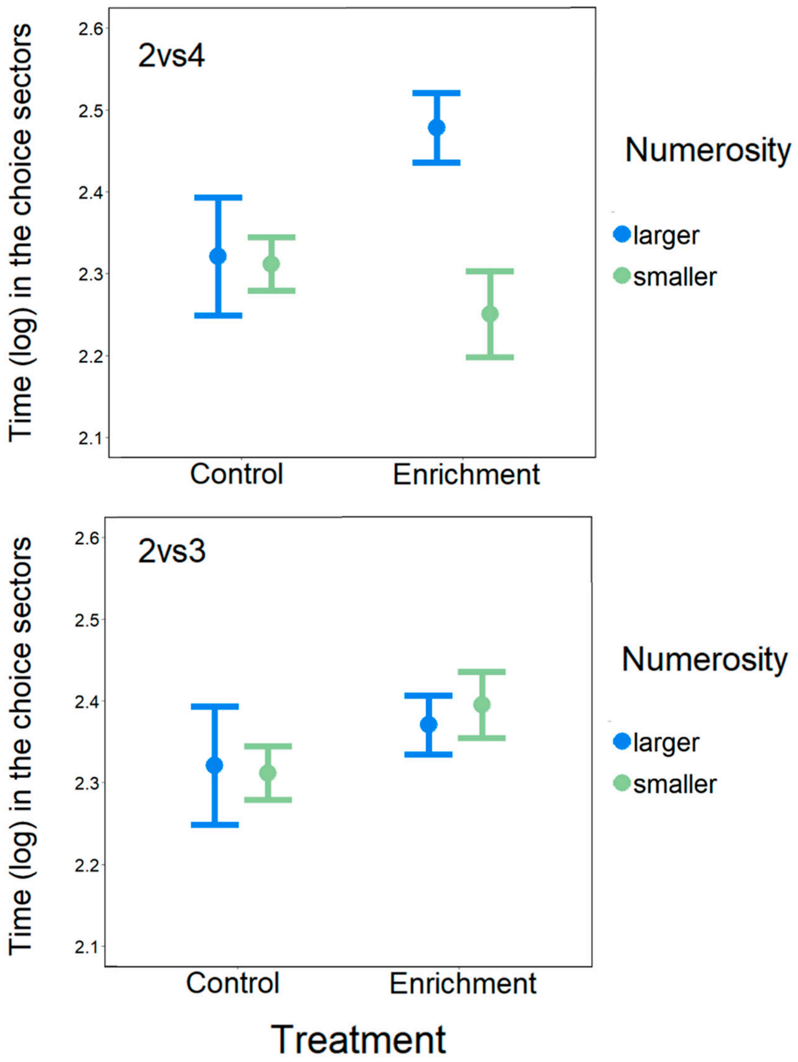

Figure S1. Number discrimination observed in Experiment 2. The Y-axis refers to the mean time (log) spent in the choice sectors with the larger and smaller numerosity for both numerical discriminations (2 vs 4 and 2 vs 3) and for both treatments (control and enrichment larvae). Bars represent the standard error
